# Correction: Immobilized Ni on TMEDA@βSiO_2_@αSiO_2_@Fe_3_O_4_: as a novel magnetic nanocatalyst for preparation of pyrido[2,3-*d*:6,5-*d*′]dipyrimidines

**DOI:** 10.1039/d3ra90048g

**Published:** 2023-06-06

**Authors:** Yasir Qasim Almajidi, Mohd Ubaidullah, Bidhan Pandit, A. K. Kareem, Rosario Mireya Romero-Parra, Adizov Bobirjon, Wesam R. Kadhum, Amran M. AL-Erjan, Munther Abosaooda, Aisha Kamal Mahmoud

**Affiliations:** a Department of Pharmacy (Pharmaceutics), Baghdad College of Medical Sciences Baghdad Iraq; b Department of Chemistry, College of Science, King Saud University P.O. Box 2455 Riyadh 11451 Saudi Arabia mtayyab@ksu.edu.sa mohdubaidullah2007@gmail.com; c Department of Materials Science and Engineering and Chemical Engineering, Universidad Carlos III de Madrid Avenida de la Universidad 30 28911 Leganés Madrid Spain; d Biomedical Engineering Department, Al-Mustaqbal University College 51001 Hillah Iraq; e Department of General Studies, Universidad Continental Lima Peru; f Chief Researcher of the Institute of General and Inorganic Chemistry, Academy of Sciences of the Republic of Uzbekistan Mirzo Ulugbek Avenue 77A 100071 Uzbekistan Tashkent; g Department of Pharmacy, Kut University College Kut 52001 Wasit Iraq; h Department of Anesthesia, College of Health & Medical Technology, Al-Ayen University Thi-Qar Iraq; i College of Pharmacy, The Islamic University 54001 Najaf Iraq; j Al-Nisour University College Baghdad Iraq

## Abstract

Correction for ‘Immobilized Ni on TMEDA@βSiO_2_@αSiO_2_@Fe_3_O_4_: as a novel magnetic nanocatalyst for preparation of pyrido[2,3-*d*:6,5-*d*′]dipyrimidines’ by Yasir Qasim Almajidi *et al.*, *RSC Adv.*, 2023, **13**, 11393–11405, https://doi.org/10.1039/D3RA01720F.

The authors regret that in the Acknowledgements section of the original manuscript, the funding information was given incorrectly.

The authors extend their sincere appreciation to the Researchers Supporting Project number (RSPD2023R682), King Saud University, Riyadh, Saudi Arabia for the support.

The Royal Society of Chemistry apologises for these errors and any consequent inconvenience to authors and readers.

## Supplementary Material

